# Subdivision of the tribe Oligaphorurini in the light of new and lesser known species from North-East Russia (Collembola, Onychiuridae, Onychiurinae)

**DOI:** 10.3897/zookeys.488.8123

**Published:** 2015-03-19

**Authors:** Anatoly B. Babenko, Arne Fjellberg

**Affiliations:** 1 Severtsov Institute of Ecology & Evolution, Russian Academy of Sciences, Leninski pr. 33, Moscow 119071; 2 Mågerøveien 168, N-3145 Tjøme, Norway

**Keywords:** Taxonomy, new species, Oligaphorurini, north-eastern Asia

## Abstract

The paper is devoted to a taxonomic review of Oligaphorurini from the north-eastern part of Palaearctic. Two new species, i.e. *Oligaphorura ambigua* **sp. n.** and *Oligaphorura duocellata* **sp. n.**, are described. Four species, *Oligaphorura nataliae* (Fjellberg, 1987), *Oligaphorura interrupta* (Fjellberg, 1987), *Oligaphorura pingicola* (Fjellberg, 1987), and *Micraphorura alnus* (Fjellberg, 1987), are redescribed on base of the types and new material, and remarks on other species known for the region, *Oligaphorura groenlandica* (Tullberg, 1876), *Oligaphorura ursi* (Fjellberg, 1984), *Oligaphorura aborigensis* (Fjellberg, 1987), and *Micraphorura absoloni* (Börner, 1901), are given to clarify their generic affiliation. Finally, merits and disadvantages of the current subdivision of the tribe are discussed and a key to the northern species of the tribe is provided.

## Introduction

Two undescribed species of the tribe Oligaphorurini from the upper reaches of Kolyma River (North-East Russia, Magadan region) do not fit the current generic subdivision of the tribe, which is mainly based on revisions made by Pomorski (1996) and Weiner (1996). Other east Palaearctic species, described by one of the authors (A. Fjellberg) from the same region, also need a critical review to fix their generic affiliation. Below, the new species are described, others are redescribed in more detail, and finally the current generic subdivision of the tribeis critically analyzed.

## The current subdivision of Oligaphorurini

Bagnall (1949) was the first author to recognize Oligaphorurinae (as a subfamily of Onychiuridae). He split it into four genera: *Archaphorura*, *Micraphorura*, *Oligaphorura* and *Dimorphaphorura* based on five species only, the sixth species, described in the same paper, is now considered as a synonym. Since then many other species have been established, often in shifting generic associations. At present, according to the database of Collembola of the World (Bellinger et al. 1996–2014) approximately 50 species of the tribe are known. Using different approaches Weiner (1996) and Pomorski (1996) retained the four original genera described by Bagnall. One new genus, *Chribellphorura*, was established by Weiner (1996) for *Onychiurus allanae* Christiansen & Bellinger, 1980, displaying a unique set of characters. However, this generic framework bears internal contradictions and does not cope with the known morphodiversity of the species. Both Pomorski and Weiner (op. cit.) based their diagnoses on the gradual reduction of the furcal field on the sternum of the fourth abdominal segment. Pomorski examined the first instar juveniles, while Weiner used adults. Both authors studied a rather limited set of mainly European species. Table 1 summarizes the diagnostic characters separating the genera and which species were involved.

Recently Shvejonkova and Potapov (2011) described three new species of Oligaphorurini which did not possess anal spines, a feature which was characteristic only to the genus *Archaphorura*. Nevertheless the species were assigned to the genera *Oligaphorura* and *Micraphorura*, an action which brings the above diagnostic scheme to a state of collapse. However, the cited authors did not establish the synonyms which would have been a natural consequence.

**Table 1.** Main diagnostic characters used for genus separation in Oligaphorurini.

		*Chribellphorura*	*Archaphorura*	*Micraphorura*	*Oligaphorura*	*Dimorphaphorura*
Weiner (1996)	Apical vesicle on *Ant*.4	present	absent			
	Tibiotarsal setae	clavate	pointed			
	Anal spines	present	absent	present		
	Furcal rudiment	finely granulated area	small pocket or finely granulated area	finely granulated area, sometimes with a kind of pocket	cuticular fold or deep pocket	finely granulated area
	Dental setae	four in line	four in two rows	two in line	four in two rows	four in line
	Manubrial setae	one row	two rows	two rows	two (seldom one) rows	one row
	Unpaired setae on *Abd*.6	*p*_0_	*m*_0_	*p*_0_ or *a*_0_ and *p*_0_	*a*_0_ and *p*_0_	*a*_0_ and *p*_0_
	Species involved	*allanae*	*serratotuberculata* and some *species that have not yet been described*	*absoloni*, *pieninensis*	*groenlandica*, *montana*, *uralica*, *judithae*, *koreana*, *linderae*	*differens*
Pomorski (1996)	Anal spines		absent	present		
	Setae on *area furcalis*		2+2 setulae + 2+2 setae	1+1 setulae + 1+1 setae	2+2 setulae + 2+2 setae	
	Species involved		*serratotuberculata*	*absoloni*, *pieninensis*	*groenlandica*, *judithae*	
Shvejonkova and Potapov (2011)	*AO*		in subapical position	normal		“*So far the independence of Dimorphaphorura calls for further ground*”
	Anal spines		absent	present or (rarely) absent		
	Distal tibiotarsal setae		11	11 or fewer		
	Furcal rudiment			cuticular furrow or finely granulated area		
	Dental setae			two or four in one row	four in two rows	
	Manubrial setae			number of rows varied		
	*Abd*.5–6		fused	separated		
Weiner and Kaprus’ (2014)	Anal spines			present	present or absent	
	Distal tibiotarsal setae			11	11	5–11
	Furcal rudiment			cuticular furrow	cuticular fold	finely granulated area
	Dental setae			1+1	2+2	absent
	*ma* setae			2 (at a level with dental setae)	2 (at a level with posterior row of dental setae)	2–4
	*mm* setae			4–6	3–6	2
	*mp* setae			4–5	4–7	4–6

In 2014 a complete revision of the genus *Dimorphaphorura* has been undertaken (Weiner and Kaprus’ 2014). The authors of this revision described six new Palaearctic species of the genus, redescribed and clarified generic affiliation of a number of other known species, and defined diagnostic characteristics of the revised genus. According to the diagnosis provided, it differs from other genera of the tribe in the organization of the furcal area (see Table 1). As a result the majority of Palaearctic species previously treated as *Micraphorura* have been transferred to *Dimorphaphorura*. The two new species described below introduce further chaos in the existing generic system.

### Abbreviations

A, AB, AC and ***ABC*** – four types of labium in Onychiuridae in accordance with the presence of thickened and blunt-tipped setae on corresponding labial papillae (Fjellberg 1999)

ABD – the fifth type of labium in Onychiuridae (Shvejonkova and Potapov 2011)

Abd.1-6 – abdominal segments

A-B, T-setae, setae M and ***Y*** – tibiotarsal setae (Deharveng 1983)

Ant.1-4 – antennal subsegments

AO – antennal organ on *Ant*.3

a0, m0, and ***p*_0_** – unpaired axial setae on terga

CNC – Canadian National Collection (Ottawa)

d0 – unpaired axial seta on *area frontalis* of the head

ma-, mm- and ***mp*- row** – anterior, medial and posterior rows of setae on manubrial field (Weiner 1996)

ms – microsensillum

MSPU – Moscow State Pedagogical University

PAO – postantennal organ

pso – pseudocellus(i)

psx – parapseudocellus(i)

q-setae – proximal setae on furcal field of Onychiuridae (Pomorski 1996)

Th.1-3 – tergal segments

## Description of new species

### Oligaphorura ambigua sp. n.

Taxon classificationAnimaliaCollembolaOnychiuridae

http://zoobank.org/36FF3284-F55E-4F97-B85C-210D66A3CBC6

Figs 1–2 , 3–9 , 29–30

#### Material.

Holotype ♂, Russia, Magadan District, upper reaches of Kolyma River, Bolshoi Annachag Mt. Range, field station “Aborigen” [61°56'N, 149°40'E], mountains above station, rather dry moss/lichen in rock crevices, 1600 m alt., 23 vii 1979, A. Fjellberg leg. (MSPU).

Paratypes 6♂, 5 ♀, and 4 juveniles, same data as holotype (MSPU); 1♂, 2 ♀, and 1 juveniles, same data but moss, lichens on rock, 1650 m alt., 23 vii 1979, A. Fjellberg leg. (MSPU).

#### Description.

**Figures 1–2.**
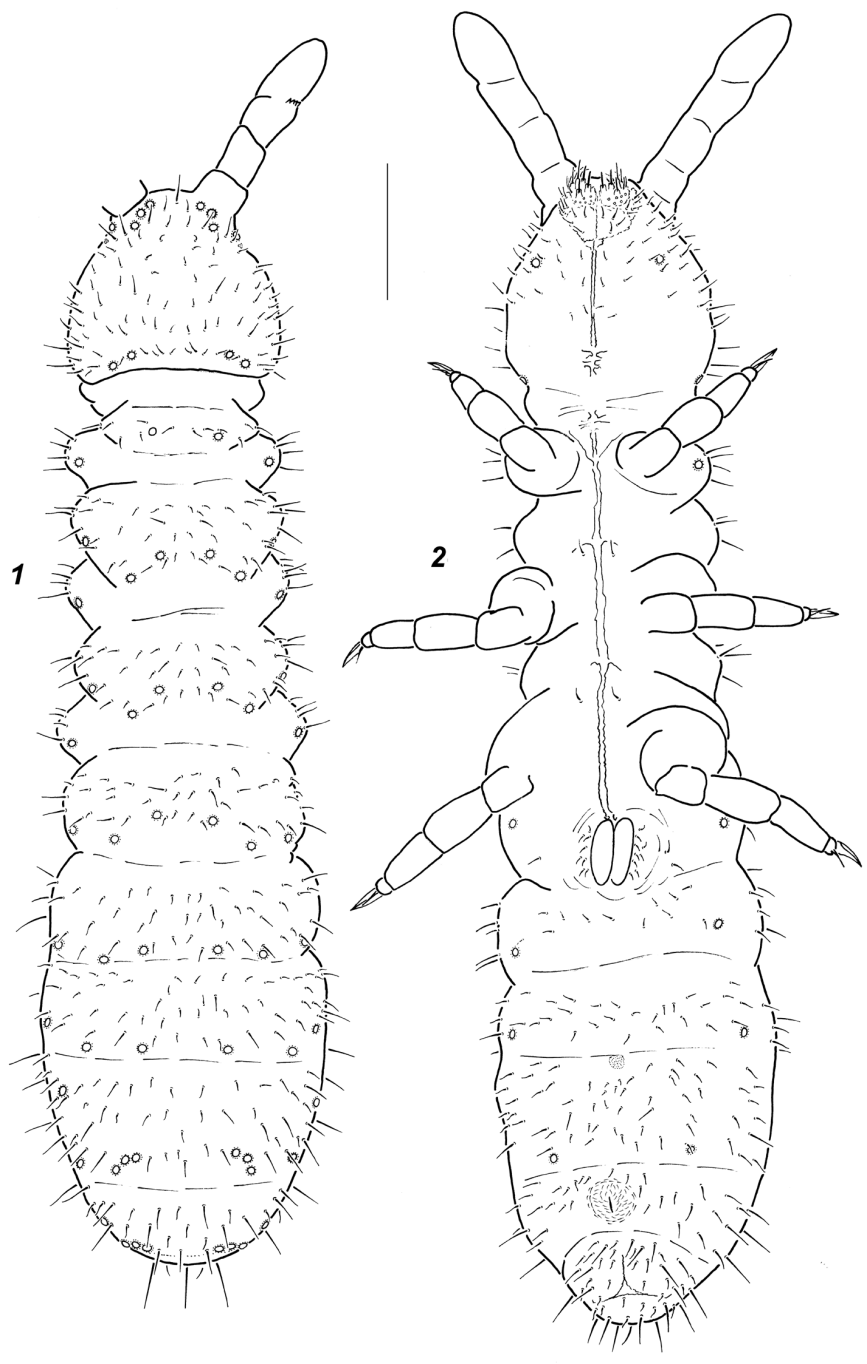
*Oligaphorura ambigua* sp. n. Chaetotaxy and *pso* position. **1** dorsal view **2** ventral view. Scale bar: 0.1 mm.

**Figures 3–9.**
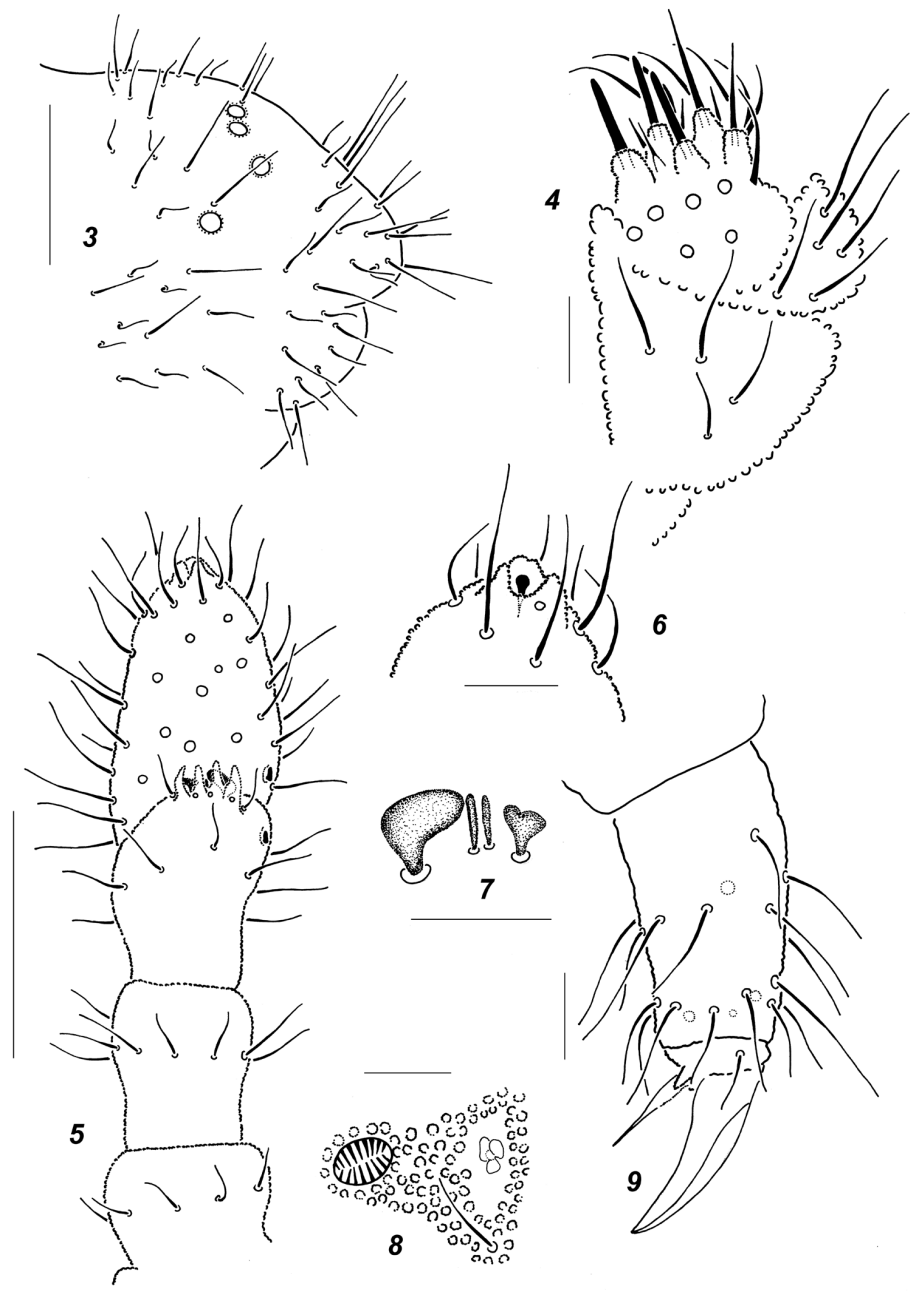
*Oligaphorura ambigua* sp. n. **3** abdominal tip **4** labium **5** antennae **6** antennal tip with subapical organite **7** sensorial elements of *AO* **8** *PAO* and nearest *pso* **9** *Ti*.3. Scale bars: **3, 5** = 0.05 mm; **4, 6–9** = 0.01 mm.

Colour white. Size of adults 0.73–0.92 mm. Body slender and elongated, *Abd*.3-4 clearly widened, *Abd*.6 short and hardly visible in dorsal view (Fig. 1), anal spines not developed (Fig. 3). Antennae about as long as head, *Ant*.4 not wider than *Ant*.3 (Fig. 5). *Ant*.4 with spherical subapical organite surrounded by cuticular papillae (Fig. 6), basal microsensillum present on level with proximal whorl of setae (Fig. 5). *AO* consisting of 4 finger-like papillae, 2 sensory rods, 2 smooth sensory clubs clearly differing in shape (Fig. 7), 5 guard setae and a lateral microsensillum (Fig. 5). *Ant*.1 and 2 with (8)9 and 14-15 setae respectively. *PAO* smaller than nearest *pso*, usually with 3 subequal lobes (Fig. 8). Labrum with 7 setae and 4 prelabral ones. Apical part of labium with thick terminal setae on papillae *A*, *B* and *C* (*ABC*-type), 7 long and 4 spiniform guard setae, and 6 proximal setae (Fig. 4). Basal fields of labium (mentum and submentum) with 4 and 5 setae, hypostomal complex of usual shape. Maxillary palp simple, with 2 sublobal setae.

Pseudocellar formulas (*pso*) as follows, dorsal: 42/133/33354, ventral: 11/000/1111, parapseudocelli (*psx*) invisible. Each upper subcoxa with two *pso*, dorsal and ventral. Localization of *pso* as in Figs 1–2. Granulation fine and uniform, without areas of enlarged granules. Dorsal chaetotaxy more or less symmetrical, with frequent variations even in axial parts of terga. Setae smooth and clearly differentiated, especially on abdominal tip: meso- and macrosetae straight, thick and blunt, microsetae curved and pointed, sensory setae indistinct (Fig. 1). *Th*.1 with 6+6 setae. Lateral *ms* present only on *Th*.2. All terga from *Th*.2 to *Abd*.3 with 3+3 axial microsetae as a rule. Unpaired dorsal seta *d*_0_ on head absent, *Abd*.4-5 usually with mesosetae *p*_0_, *Abd*.6 dorsally with 1-2 axial macrosetae. Thoracic sterna of *Th*.2-3 with 1+1 setae along ventral line, rarely absent on one or both sterna, ventral chaetotaxy of abdomen as in Fig. 2. Furca reduced to a small area of fine granulation situated at contact with borders of *Abd*.3-4 sterna, with 2+2 small posterior setae arranged in 2 rows and surrounded by several (age dependent) longer setae including two flank macrosetae in row *mp* (*cf.* Fig. 29 and Fig. 30). Ventral tube with (8)9+9 distal setae and 1(2) proximal ones at base. Upper subcoxae usually with 4-5-5, tibiotarsi with 20-21-20 setae as a rule. Distal whorl with 11 setae (7 *A* and 4 *T*-setae), whorl *B* with 7-7-6 setae, setae *M* and 1-2 setae of *C*-whorl present. Unguis simple, with neither inner nor lateral teeth, unguiculus without distinct basal lamella, clearly shorter than unguis (Fig. 9).

#### Affinities.

This new species resembles two congeners recently described from the European part of Russia, namely *Oligaphorura humicola* Shvejonkova & Potapov, 2011 and *Oligaphorura kremenitsai* Shvejonkova & Potapov, 2011. All three species lack anal spines and have no cuticular fold on the sternum of *Abd*.4. Apart from this, the former is characterized by a set of *pso* on both dorsal and ventral sides on a body, as well as on upper subcoxae identical to that in *Oligaphorura ambigua* sp. n., and also has no *ms* on *Th*.3 and ventral *psx*. *Oligaphorura kremenitsai* differs from both *Oligaphorura ambigua* and *Oligaphorura humicola* having more *pso* on *Th*.2-3 (42/144/33354 as a whole). Some differences like the uncommon position of anterior *pso* on head and submedial ones on *Abd*.4-5 in *Oligaphorura humicola* and *Oligaphorura kremenitsai* or their reduced tibiotarsal chaetotaxy may be a result of small body size (< 0.6 mm). Nevertheless, the palp structure (*ABD*-type) in *humicola*/*kremenitsai* and the loss of labial papilla *C* probably reflects a certain genetic distance.

The presence of only four papillae in *AO* is also an unusual condition in the tribe, shared only with *Micraphorura absoloni* (Börner, 1901), *Oligaphorura palissai* (Yosii, 1971) and *Dimorphaphorura sophyae* Weiner & Kaprus’, 2014. The clear cuticular papillae on antennal tip are also quite characteristic.

#### Etymology.

The name reflects the uncertain generic position of the new species.

#### Distribution.

Known only from the type locality, the alpine belt in the upper reaches of Kolyma river.

### Oligaphorura duocellata sp. n.

Taxon classificationAnimaliaCollembolaOnychiuridae

http://zoobank.org/D3CCBC1E-3F6C-4737-A999-13C07A501D45

Figs 10–16

#### Material.

Holotype ♂, Russia, Magadan District, upper reaches of Kolyma River, Bolshoi Annachag Mt. Range, field station “Aborigen” [61°56'N, 149°40'E], mosses on slope, 1400-1500 m alt., 27.vii.1979, leg. A. Fjellberg (MSPU).

Paratypes 1♂, 1♀, and 1 juvenile, same data as holotype (MSPU).

#### Description.

Colour white. Size of adults 1.2–1.3 mm. Body slender and elongated. Antennae slightly shorter than head, club-like with *Ant*.4 clearly wider than *Ant*.3. Subapical organite on *Ant*.4 peg-like, basal microsensillum set on level with proximal whorl of setae. *AO* consisting of 5 long papillae, 2 sensory rods, 2 granulated sensory clubs clearly differing in shape (Fig. 12), 5 guard setae and a lateral microsensillum. *Ant*.1 and 2 usually with 8 and 14 (15) setae, respectively. *PAO* about as long as nearest *pso*, usually with 3 elongated lobes (Fig. 11). Labrum with 9 setae and 4 prelabrals. Apical part of labium with thick terminal setae on papillae *A* and *C* (*AC*-type), terminal setae on all papillae rather short (Fig. 13), 7 long, usual 4 spiniform guard setae and 6 proximal setae present. Basal fields of labium (mentum and submentum) with 4 and 5 setae, hypostomal complex of usual shape. Maxillary palp simple, with 2 sublobal setae.

**Figures 10–16.**
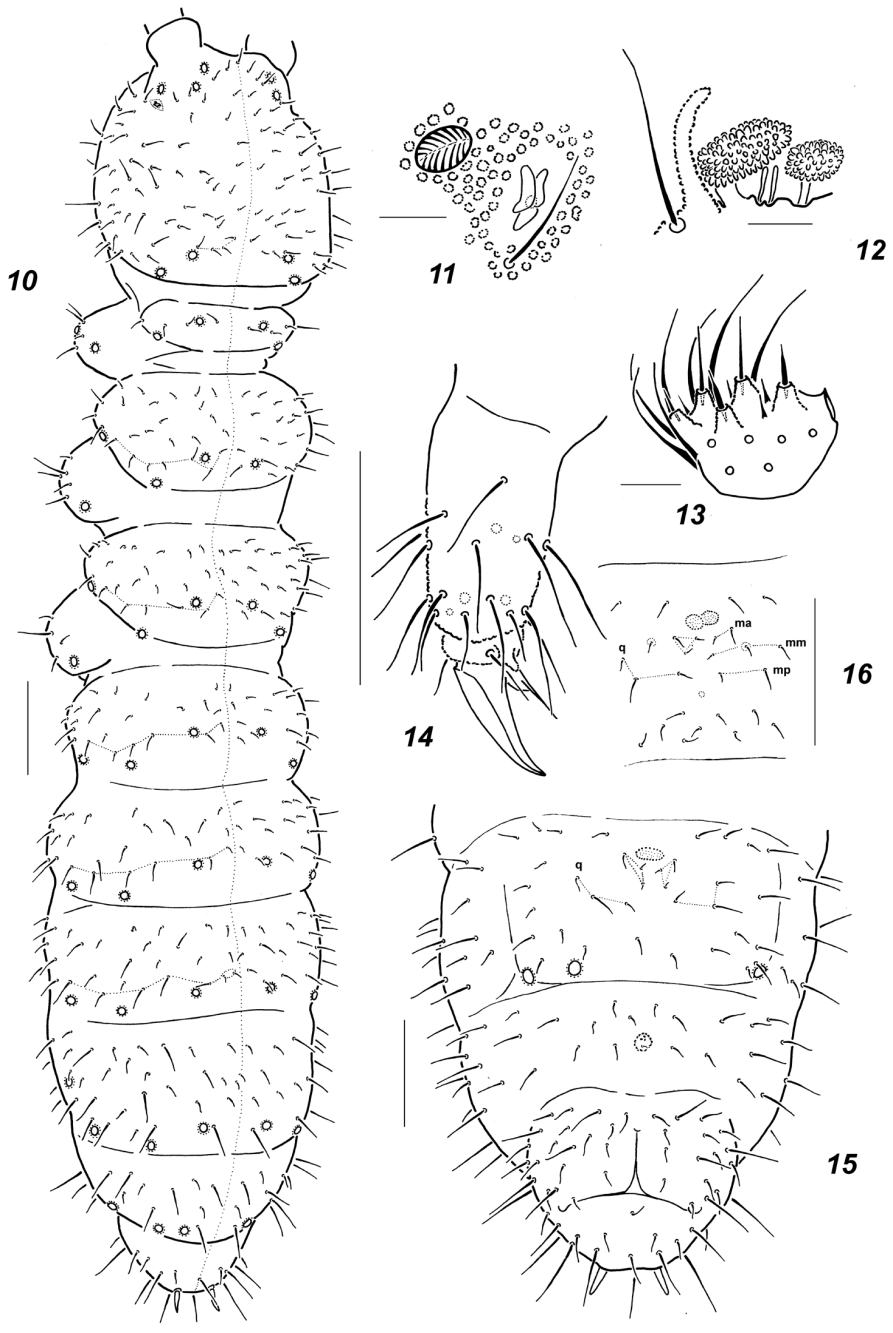
*Oligaphorura duocellata* sp. n. **10** dorsal chaetotaxy and *pso* position **11** *PAO* and nearest *pso* **12** sensorial elements of *AO* **13** labium **14** *Ti*.2 **15** chaetotaxy of abdominal sterna, juvenile **16** ibid, adult. Scale bars: **10, 16** = 0.1 mm; **32, 15** = 0.05 mm; **11–13** = 0.01 mm.

Pseudocellar formulas (*pso*) as follows, dorsal: 32/(1)233/33343, ventral: 11/000/1111(2), parapseudocelli (*psx*) hardly visible (probably due to long preservation), but *psx* on unpaired anal lobe present. Upper subcoxae with 2-(2)3-3 *pso*, one dorsal and 1-2 ventral. Localization of dorsal *pso* as in Fig. 10, submedial *pso* on *Abd*.4 set far apart. Granulation fine and uniform, without areas of enlarged granules. Dorsal chaetotaxy more or less symmetrical, but with frequent variations even in axial parts of terga. Setae smooth and clearly differentiated only on abdominal tip: meso and macrosetae straight, thick and blunt, microsetae curved and pointed, sensory setae indistinct (Fig. 10). *Th*.1 with few setae, (3)4+4 as a rule, even in full grown specimens. Both *Th*.2 and 3 with lateral *ms*. All terga from *Th*.2 to *Abd*.3 with 3+3 axial microsetae as a rule. Setae *p*_1_ set clearly in forward position comparing with *p*_2_ setae on head and *Th*.2-*Abd*.3. Unpaired dorsal seta *d*_0_ on head absent, *Abd*.4 *m*_1_ setae fine and curved, much shorter than straight *a*_1_ and *p*_1_, *Abd*.6 with axial macroseta *a*_0_ almost subequal to *a*_2_ setae. Thoracic sterna of *Th*.2-3 without setae along ventral line. Furca reduced to a small area with fine granulation situated in some distance from anterior border of *Abd*.4, 2+2 setae arranged in 2 rows below furcal remnant are clearly shorter then surrounded ones and moved posteriorly (Fig. 16). In juveniles manubrial field with 3+3 setae between furcal remnant and *q*-setae (Fig. 15), adult with few additional setae in intermediate position (Fig. 16). Ventral tube with 7+7 distal setae and 1-2 proximal ones at base. Upper subcoxae usually with 3-4-(4)5 setae, tibiotarsi with more than 20-20-19 setae: distal whorl always with 11 setae (7 *A* and 4 *T*-setae), whorl *B* with 7-7-6 setae, setae *M* and variable *C*-whorl with one or two setae present. Unguis simple, with neither inner nor lateral teeth, unguiculus with wide basal lamella, clearly shorter than unguis (Fig. 14). Anal spines long and rather thin set without clear papillae.

#### Affinities.

Several uncommon features, like 2+2 pseudocelli and few setae on *Th*.1, the presence of pseudocelli on several abdominal sterna, the absence of setae on thoracic sterna, and a furcal remnant in the form of a finely granulated area with 4 small setae behind it, permit easy identification of the new species. In addition to *Oligaphorura duocellata* sp. n. nine known species of the tribe possess pseudocelli on several abdominal sterna. Three of them, *Archaphorura serratotuberculata* (Stach, 1933), *Archaphorura alavensis* Simón & Luciáñez, 1994, and *Archaphorura marcuzzii* (Cassagnau, 1968) are usually considered as representatives of the genus *Archaphorura* due to the absence of anal spines. The presence of ventral *pso* in the former species is uncertain as specimens from Moscow vicinity contrary to those from Poland (see Pomorski 1998) have only ventral *psx* on abdomen. The loss of anal spines also characterizes *Oligaphorura humicola*, *Oligaphorura kremenitsai* and *Oligaphorura ambigua* sp. n.

The only known species of the tribe with *AS* and *pso* on several abdominal sterna (*Micraphorura multiperforata* (Gruia, 1973), *Micraphorura uralica* (Khanislamova, 1986), are within *Micraphorura* on the www.collembola.org or treated as *Dimorphaphorura* (*Dimorphaphorura olenae* Weiner & Kaprus’, 2014). *Micraphorura multiperforata* is a unique species with dorsal *pso* multiplication, whereas *Micraphorura uralica* seems to be the most similar to *Oligaphorura duocellata* sp. n. having also more than 2 *pso* on subcoxae (a unique character) and no setae on thoracic sterna, a character which is known only for species from eastern parts of Asia and North America, i.e. *Oligaphorura nuda* (Fjellberg, 1987), *Oligaphorura judithae* (Weiner, 1994), *Oligaphorura linderae* (Weiner, 1994), *Oligaphorura montana* Weiner, 1994, *Oligaphorura pseudomontana* Sun & Wu, 2012, and *Oligaphorura chankaensis* Sun & Wu, 2012.

#### Etymology.

Named after the presence of 2+2 *pso* on *Th*.1, a character previously unknown for the tribe.

#### Distribution.

Known only from the type locality.

### Redescription and remarks on other species of Oligaphorurini from north-east Russia

#### Micraphorura absoloni

Taxon classificationAnimaliaCollembolaOnychiuridae

(Börner, 1901)

Aphorura absoloni Börner, 1901: 422.
Micraphorura absoloni (Börner): www.collembola.org

##### Remarks.

Juveniles from Magadan (NE Russia) have a furcal field with 2+2 setae behind the cuticular furrow, followed by 3+3 *q*-setae (Fig. 27). This pattern is in a full accordance with what Pomorski (1996) reported from European populations. In adults a few additional setae (usually in asymmetric positions) may appear between the primary rows of the juvenile, obscuring the original pattern (Fig. 28). The formula of the parapseudocelli (*psx*) in specimens from Magadan is also the same as Pomorski (1998) noticed from Europe: 0/000/1101, absent on subcoxae.

#### Oligaphorura nataliae

Taxon classificationAnimaliaCollembolaOnychiuridae

(Fjellberg, 1987) comb. n.

Onychiurus (Archaphorura) nataliae Fjellberg, 1987: 281.
Micraphorura nataliae (Fjellberg): www.collembola.org

##### Material.

holotype, ♂, “USSR, Chukotka, Chaun Bay [68°44'N, 170°36'E], upland heath, soil, 13.viii 1977” (CNC 165046, type No 20114); paratypes: 1 juv. same data (CNC 165136, type No 20113); 3♀, same place, Loc. S-1, Sept. 1975 (CNC 165135, type No 20112), all S.F. MacLean leg.

##### Additional material.

15 specimens, Russia, Novosibirsk Islands, Kotel’nyi, Balyktakh river [75°03'N, 140°10'E], various habitats, vii 1994, A. Babenko leg.

##### Redescription.

Colour white. Size 0.8–0.9 mm. Body shape cylindrical. Antennae about as long as head, *Ant*.3-4 broad, club-like. *Ant*.4 with subapical organite and microsensillum located in proximal row of setae. *AO* consists of 5 long and thin papillae, two sensory rods, two granulated sensory clubs (internal straight, external much bigger and bent), 5 guard setae, and a lateral microsensillum which is set below the organ. *Ant*.1 and 2 with 8 and 14-15 setae, respectively. *PAO* with 3-4 lobes, slightly longer than nearest pseudocellus. Labrum with 4/5-2-2 setae but variations also seen. Apical part of labium with thick terminal setae on papillae *A* and *C*, usually complete number of long guard setae (7) and 4 spiniform ones, 6 proximal setae present. Basal fields of labium with 4+5(6) setae. Maxillary palp simple with two sublobal hairs. Maxillae not modified.

Pseudocellar formula (*pso*) as follows, dorsal: 32/033/33343, ventral: 2/000/0000, parapseudocelli (*psx*) invisible. Each subcoxa with one *pso*, *psx* invisible (absent ?). Granulation fine, clearly coarser around pseudocelli on all segments. Dorsal chaetotaxy almost symmetrical, setae smooth, macrosetae clearly differentiated only on abdominal tip, sensory setae indistinct. Th.1 with (5)6+6 setae. Lateral *ms* present only on *Th*.2. On head *p*_1_ clearly above *p*_2,_ its position on *Th*.2-*Abd*.3 rather variable but usually more or less at a level with *p*_2_. *Abd*.1-3 with setae *p*_4_ present as a rule. *Abd*.5 with *m*_1_ curved, thinner and shorter than the straight *a*_1_ and *p*_1_, the latter usually shorter than anterior macrosetae *a*_1_. Unpaired setae: *d*_0_ absent, *Abd*.5 often with seta *p*_0_ present, *Abd*.6 with two axial macrosetae, *a*_0_ clearly shorter than *a*_2_. Thoracic sterna with 0, 1+1, 1+1 setae. Furca as small cuticular furrow in some distance from anterior border of sternum. Chaetotaxy of furcal field in juveniles as in Fig. 32: usual 3+3 proximal *q*-setae and 3+3 setae set in triangle below furrow; adults with few (1-2) additional setae in front of *q*-row (Fig. 31). Ventral tube usually with 6+6 distal and 2(1) proximal setae at base. Subcoxae with 3-5-(4)5 setae, tibiotarsi with 20-20-19 setae: each distal whorl (*A+T*) with 11 setae, whorl *B* with 7-7-6 setae, setae *M* and *Y* present on all tibiotarsi. Unguis simple, without inner or lateral teeth, unguiculus with small basal lamella, about 3/4 as long as unguis. Anal spines rather long and thin, almost straight and hardly constricted at base, set without papillae. Males present.

##### Remarks.

Originally described as Onychiurus (Archaphorura) nataliae, the species is now listed under *Micraphorura* on www.collembola.org. Nevertheless the chaetotaxy of the manubrial field in juveniles of this species is identical with that found in northern parthenogenetic populations of *Oligaphorura groenlandica* (Tullberg, 1876) (*cf.* Fig. 32 and Fig. 18). Adults usually have a pattern with four setal rows behind the cuticular furrow (Fig. 31), which considered being typical for *Oligaphorura*.

**Figures 17–20.**
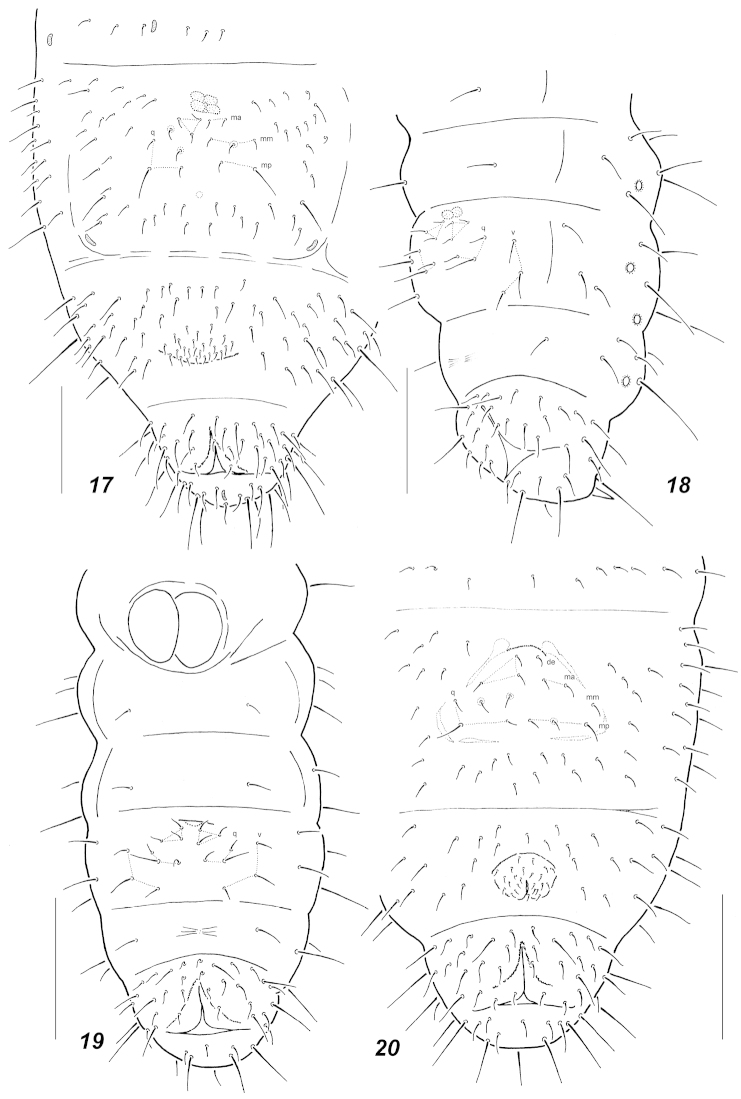
Chaetotaxy of abdominal sterna. **17** *Oligaphorura groenlandica* (adult, Taimyr) **18** *Oligaphorura groenlandica* (I instar, Taimyr) **19** *Oligaphorura interrupta* (I instar) **20** *Oligaphorura interrupta* (adult). Secondary setae in adults circled. Scale bars: **17** = 0.1 mm, **18–20** = 0.05 mm.

In the interactive key on www.collembola.org the species keys out with *Oligaphorura interrupta* (*Micraphorura* on www.collembola.org) which can easily be distinguished by higher number of abdominal *pso*, presence of *ms* on *Th*.3 and absence of ventral setae on *Th*.2.

Five known species of the tribe possess the same number of dorsal and ventral pseudocelli as *nataliae*, i.e. *Oligaphorura pingicola* (Fjellberg, 1987), *Oligaphorura koreana* (Weiner, 1994), *Dimorphaphorura raxensis* (Gisin, 1961), *Dimorphaphorura chatyrdagi* (Kaprus’, Weiner & Pomorski, 2002), and *Dimorphaphorura sanjiangensis* Sun & Wu, 2012. *Oligaphorura nataliae* differs from the above *Oligaphorura* species (*Oligaphorura pingicola* and *Oligaphorura koreana*) in having no *ms* on *Th*.3. *Dimorphaphorura raxensis* according to Weiner and Kaprus’ (2014) has 9 distal setae on tibiotarsi and *ABC* type of labium. *Dimorphaphorura chatyrdagi* can easily be distinguished due to strongly reduced tibiotarsal chaetotaxy (with 5 distal setae) and the presence of *ms* on *Th*.3. *Dimorphaphorura sanjiangensis*, recently described from northern China, can be separated from *Oligaphorura nataliae* due to different type of labium (*A* versus *AC* in *Oligaphorura nataliae*), the presence of *psx* on abdominal sterna (0/000/122201+1^m^), and identical number of tibiotarsal setae on all legs (20-20-20) which is very characteristic if it is correct. Apart of this, all three species of *Dimorphaphorura* should have no more than 5+5 setae in the manubrial field whereas even first instars of *Oligaphorura nataliae* possess 6+6 setae.

The presence of 7 long guard setae of labium in such small species as *Oligaphorura nataliae* is an uncommon character in the tribe and needs additional confirmation being seen in few specimens. Only five other Asiatic species, *Dimorphaphorura sanjiangensis*, *Micraphorura changbaiensis* Sun & Wu, 2012, *Oligaphorura aborigensis* (Fjellberg, 1987) and the two new *Oligaphorura* species described above, share this character with *Oligaphorura nataliae* whereas 18 species of the tribe are known as having only 6 long guards.

#### Micraphorura alnus

Taxon classificationAnimaliaCollembolaOnychiuridae

(Fjellberg, 1987) comb. n.

Onychiurus (Archaphorura) alnus Fjellberg, 1987: 282.
Dimorphaphorura alna (Fjellberg): www.collembola.org
Dimorphaphorura alnus (Fjellberg): Weiner and Kaprus’ 2014: 6.

##### Material.

holotype, ♀, “ USSR, Magadan Reg., Aborigen [field station, 61°56'N, 149°40'E], deep, moist *Pinus pumila* litter, 27 vii 1979“ (CNC 165044, type No 20108); Paratypes, 5 ♀, same sample (CNC 165130, type No 20111); 1 ♀, same region, “*Alnus* litter in dense thickets, 25 vii 1979” (CNC 165129, type No 20110), all A. Fjellberg leg.; 9 specimens (in bad condition), “USSR, Chukotka, Chaun Bay [68°44'N, 170°36'E], Sept. 1975” (CNC 165128, type No 20109), S.F. MacLean leg.

##### Additional material.

1 ♂, Magadan District, Bolshoi Annachag Mts. Range, upper reaches of Kolyma River, field station “Aborigen”, valley bottom near station, moss and litter of *Larix*/*Pinus* on slope, 25 vii 1979, A. Fjellberg leg.; 5♀ and 3♂, same region, deep moist litter in thickets of *Pinus pumila*, 1200 m alt., 27 vii 1979, A. Fjellberg leg.; 2 ♂, same region, stand of *Pinus pumila*, *Betula*, *Larix dahurica*, and *Alnus fruticosa*, 24 vii 1979, V. Behan leg.

##### Redescription.

Colour white. Size 0.8-0.9 mm. Body shape cylindrical. Antennae about as long as head, *Ant*.3-4 broad, club-like. *Ant*.4 with subapical organite and microsensillum located in proximal row of setae. *AO* consists of 5 long and thin papillae, two sensory rods, two granulated sensory clubs (internal almost straight, external much larger and bent), 5 guard setae and a lateral microsensillum which is set below the organ. *Ant*.1 with 8 setae, Ant. 2 with (12)13 setae. *PAO* with 3-4 lobes, slightly longer than nearest pseudocellus. Labrum with 4/3-2-2 setae. Apical part of labium with thick terminal setae on papillae *A* and *C*, 6 long guard setae and 6 proximal setae present, basal fields with 4+5 setae. Maxillary palp simple with two sublobal hairs.

Pseudocellar formula (*pso*) as follows, dorsal: 32/133/33343, ventral: 2/000/0001, parapseudocelli (*psx*) invisible. Each subcoxa with one *pso*, *psx* absent. Granulation rather coarse, especially around pseudocelli and on *Abd*.6. Dorsal chaetotaxy almost symmetrical, setae smooth and fine, macrosetae poorly differentiated, sensory setae (2/011/222211) more or less distinct, *Th*.1 usually with 6+6 setae, *Th*.2-3 with lateral *ms*, *p*_1_ on head and *Th*.2-3 almost on level with other medial *p*-setae. *Abd*.5 with *m*_1_ longer than *a*_1,_ subequal to *p*_1_. Unpaired setae: *d*_0_ and axial seta on *Abd*.5 absent, *Abd*.6 with two axial setae, *a*_0_ subequal to *a*_2_. Thoracic sterna with 0-1(2)-1(2-3) setae on each side of ventral line. Upper subcoxae usually with 3-3-4 setae. Furca as a small area with fine granulation in middle section of sternum of *Abd*.4, some setae present on sternum anteriorly to furcal remnant. In juveniles manubrial field with usual 3+3 proximal *q*- setae and 2+2 distal ones set in a row, in adults some additional setae sometimes present, especially in large specimens (Fig. 33). Ventral tube with (5)6+6 distal and (1)2 proximal setae at base. Subcoxae with 3-(3)4-(3)4 setae, tibiotarsi with 20-20-19 setae: each distal whorl (*A+T*) with 11 setae, whorl *B* with 7-7-6 setae, setae *M* and *Y* present on all tibiotarsi. Unguis simple, without inner or lateral teeth, unguiculus with clear basal lamella, about 3/4 as long as unguis. Anal spine bent, rather thick and constricted at base, set without papillae. Males present.

##### Remarks.

The redescription completely matches the original one, although Fjellberg (1987) did not mentioned ventral *pso* on *Abd*.4. The species was recently redescribed by Weiner and Kaprus’ (2014). The only clear difference with this description is the number of labral setae which states as being full (4/3-4-2). The authors treat the species as *Dimorphaphorura* because their specimens had no secondary setae in the manubrial field (only 5+5 setae in all). In the largest specimens from Magadan the manubrial field has several additional *mm*-setae (the holotype has 14 setae on the manubrial field in total) which illustrates the weak distinction between *Dimorphaphorura* and *Micraphorura*.

The set of dorsal pseudocelli displayed by *Micraphorura alnus* is shared with several other species in the genus. Among these only four species have ventral *pso* present on *Abd*.4, i.e. *Micraphorura alnus*, *Micraphorura pieninensis* Weiner, 1988, *Dimorphaphorura irinae* (Thibaud & Taraschuk, 1997), and *Dimorphaphorura olenae*. *Micraphorura pieninensis* differs from both other species in having no *ms* on *Th*.3. *Dimorphaphorura irinae* can be distinguished by the reduced tibiotarsal chaetotaxy (only 2 *T*-setae present), a full number of labral setae (4/9) and different labial type (*ABC*) (Weiner and Kaprus’ 2014). *Dimorphaphorura olenae* possesses ventral *pso* on all sterna from *Abd*.1 to *Abd*.4, and two *pso* on subcoxae of leg.2-3.

#### Oligaphorura interrupta

Taxon classificationAnimaliaCollembolaOnychiuridae

(Fjellberg, 1987) comb. n.

Onychiurus (Archaphorura) interruptus Fjellberg, 1987: 282.
Micraphorura interrupta (Fjellberg): www.collembola.org

##### Material.

holotype, ♀, “USSR, Magadan Reg., “Death Valley”, Magadan-Ust’ Umchug [road], 209 km from Magadan, moss, lichen, *Vaccinium*, 30 vii 1979” (CNC 165045, type No 20107); Paratypes, 9♀ and 1♂, same sample (CNC 165134, type No 20106), all A. Fjellberg leg.

##### Additional material.

1 specimen, Magadan District, Bolshoi Annachag Mts. Range, upper reaches of Kolyma River, field station “Aborigen” [61°56'N, 149°40'E], alpine study area (lichen, moss, *Dryas*, *Empetrum*), 26 vii 1979, V. Behan leg.; 11 specimens, same region, Butugychag (“Death Valley”) [61°18'N, 149°11'E], moist *Sphagnum*, litter *Betula nana*, *Alnus* thickets, 30 vii 1979, A. Fjellberg leg.; 10 specimens, Magadan vicinities, “Snow Valley”, rich meadow (*Veratrum*, *Angelica*), 20 viii 1979; ca. 15 specimens, Northern Yakutia, Shirokostan Peninsula, Ledyanoe lake [72°25'N, 141°00'E], various habitats, 1994, A. Babenko leg.; 1 ♀, North-Eastern Yakutia, delta of Indigirka river [71°26'N, 149°45'E], *Eriophorum vaginatum* tussock, 1994, A. Babenko leg.; ca. 30 specimens, Magadan District, upper reaches of Ola River [60°39'N, 151°16'E], various sites, viii 2011, A. Babenko leg.

##### Redescription.

Colour white. Size 0.75 mm. Body shape cylindrical. Antennae about as long as head, *Ant*.3-4 broad, club-like. *Ant*.4 with a peg-like subapical organite, microsensillum located in proximal row of setae. *AO* consisting of 5 long and thin papillae, two sensory rods, two granulated sensory clubs (internal straight, external much bigger and bent), 5 guard setae and a lateral microsensillum set below the organ. *Ant*.1 and 2 with 8 setae and 13-14 setae respectively. *PAO* with 3-4 lobes, longer than nearest pseudocellus. Labrum with 4/3-2-2 setae. Apical part of labium with thick terminal setae on papillae *A* and *C*, common number of guard setae (6 long and 4 spiniform ones), and 6 proximal setae. Basal fields with 4+5(6) setae. Maxillary palp simple with two sublobal hairs.

Most common dorsal pseudocellar formula (*pso*) as 32/033/33353, submedial *pso a* and *b* on *Abd*.1-2 set close together (with *pso b* on level with setae *p*3). Variations are frequent and specimens with additional *pso* on some abdominal terga (usually asymmetrical) are seen. The whole formula may be expressed as follows, 32/033/3(4),3(4),3(4),(4)5,3(4). Ventral side of head with two *pso* as usual. Parapseudocelli (*psx*) invisible. Each subcoxa with one *pso*. Granulation fine and uniform, sometimes clearly coarser around pseudocelli. Dorsal chaetotaxy almost symmetrical, setae smooth, macrosetae short, needle-like usually blunt at tip, sensory setae more or less distinct, usually 2/011/22211 in number, sensilla like, broaden seta usually present on lower *Scx*.3. *Th*.1 with 5-6 setae on each side. Both *Th*.2 and 3 with lateral *ms*, *p*_1_ on head and *Th*.2-3 usually slightly in front of *p*2, *Abd*.1-3 with setae *p*4 usually present. *Abd*.5 with *m*_1_ curved, thinner and shorter than the straight *a*_1_ and *p*_1_. Unpaired setae: *d*_0_ absent, *p*_0_ frequently present on *Abd*.5, *Abd*.6 with two axial setae, *a*_0_ clearly shorter than *a*_2_. Thoracic sterna 1-3 with 0-0-1 setae, sometimes setae completely absent. Furca remnant as a small fold in some distance from anterior border of *Abd*.4 sternum, chaetotaxy of manubrial field in juveniles as in Fig. 19, usually with 3+3 proximal *q*- setae and 3+3 setae in triangles between cuticular fold and *q*- setae. In adults 1-3 additional setae usually present in intermediate position (Fig. 20). Four irregular setal rows may be distinguished. Ventral tube with 6+6(7) distal and 2(1) proximal setae at base. Subcoxae usually with 3(4)-4(5)-4 setae, tibiotarsi with 20-20-19 setae: each distal whorl (*A+T*) with 11 setae, whorl *B* with 7-7-6 setae, setae *M* and *Y* present on all tibiotarsi. Unguis simple, without inner or lateral teeth, unguiculus with small basal lamella, about 3/4 as long as unguis. Anal spines short and thick, slightly bent and constricted at base, set without papillae.

##### Remarks.

The number of pseudocelli in the species appears to be more variable than stated in the original description by Fjellberg (1987), even within the region of the type locality. That is why Nearctic *Oligaphorura nuda*, characterized by increased number of abdominal *pso*, appears to be hardly separable from *Oligaphorura interrupta* despite their different generic positions on the www.collembola.org. Nevertheless the chaetotaxy of manubrial field in *Oligaphorura interrupta* is identical to that of *Oligaphorura groenlandica* (*cf.* Figs 17–18 and Figs 19–20) and clearly differs from the pattern typical of *Micraphorura absoloni* (Figs 26–27). The structure of manubrial field in the *nuda* holotype (CNC 165047, type No 20103) also indicates its position within the genus *Oligaphorura*.

The presence of ventral setae on *Th*.3 in *Oligaphorura interrupta* was used by Fjellberg (1987) as an additional diagnostic character to separate *Oligaphorura interrupta* and *Oligaphorura nuda* (setae absent). However, new material of *Oligaphorura interrupta* from various regions of eastern Palaearctic shows this character to be invalid. Some specimens of *Oligaphorura interrupta* may also be completely devoid of ventral setae on thorax. More material is evidently needed to clarify the real relationships between these two species.

Only one other known species of the tribe shares the absence of *pso* on *Th*.1 combined with presence of 5 *pso* on *Abd*.4 with *Oligaphorura interrupta* and *Oligaphorura nuda*: *Oligaphorura reversa* (Fjellberg, 1987). This characteristic species differs from the above-mentioned species in having an unusual position of the dorsal pseudocelli on *Abd*.1-3: the medial *pso a* is set in a posterior position, clearly behind submedial *pso b*.

The species listed as Oligaphorura sp. aff. nuda in Babenko (2013) from Taimyr is another congener with a similar dorsal pseudocellar formula. It differs from both *Oligaphorura interrupta* and *Oligaphorura nuda* having 1+1 ventral *pso* on *Abd*.4.

#### Oligaphorura groenlandica

Taxon classificationAnimaliaCollembolaOnychiuridae

(Tullberg, 1876)

Lipura groenlandica Tullberg, 1876: 41.
Oligaphorura groenlandica (Tullberg): www.collembola.org

##### Remarks.

Pomorski’s (1996) description of the furcal area of the first instar was as follows: …*q-chaetotaxy – 3 chaetae, area furcalis with 2+2 setulae below cuticular furrow and 2+2 setae at base* [all together 4+4 setae]. It was based on a single specimen from a bisexual population from Wolin Island on the Polish shore of the Baltic Sea. Weiner’s description (1996): …*small, finely granulated cuticular fold or quite a deep pocket with 2 setae on its posterior edge, sometimes with 1+1 additional setae and two other dental setae posteriorly, with manubrial setae on both sides and with other manubrial setae usually in two rows* is more complicated. According to the interactive key on the www.collembola.org *Oligaphorura* should have two dental setae on the fold or posteriorly and three manubrial rows of setae behind them. In fact, the type species of the genus, *Oligaphorura groenlandica* (or more correctly the most common parthenogenetic form of this species) has no cuticular fold or clear furrow, just an area with fine granulation in anterior third of the sternum of *Abd*.4 (Fjellberg 1998). In adults, the position of setae of the furcal area is rather irregular due to weak polychaetosis (Fig. 17) and juveniles clearly differ from that described by Pomorski with only 3+3 setae in front of the 3+3 *q*-setae (Fig. 18).

Unfortunately, this parthenogenetic form is not the only one present in the northern areas of the Palaearctic. On Taimyr Peninsula and Novosibirsk Islands another bisexual form was found. Probably the same (or similar) form exists in southern Norway (Fjellberg 1998) and Poland (Pomorski 1998). Its furcal area is more similar to the described pattern for *Oligaphorura groenlandica* by Pomorski (1996) with cuticular fold and 4+4 setae between the fold and *q*-setae in the first instar juveniles (Fig. 26). In adults, two “dental” setae set in front of three irregular manubrial rows of setae (Fig. 25). These two forms are very similar and apart from the furcal area, differ only in size (the parthenogenetic form is larger) and in differentiation of the medial setae on *Abd*.5: “microsetae” *m*_1_ (thin and pointed) are much longer than macrosetae *a*_1_ and *p*_1_ (straight and truncate) in the true parthenogenetic *Oligaphorura groenlandica*. Bisexual specimens usually have *m*_1_ curved and short and *a*_1_ and *p*_1_ long and straight. There are also some differences in *psx* formulas: 10/000/222201+1^m^, upper subcoxae with 2-2-2 *psx* in the bisexual form and 10/000/222101^m^, subcoxae 1-1-2 in the parthenogenetic one. Unfortunately the number of *psx* in the latter form is not stable. Some specimens lack postlabial *psx* or one of *psx* on anterior abdominal sterna, others may have additional *psx* on *Abd*.4 or on paired anal lobes; and anterior *psx* on subcoxae of fore and middle legs can be just invisible due to position. Pomorski (1998) gave slightly different formula for the Polish specimens: 1/000/122101^m^. Thus several similar forms do exist in Palaearctic, but the real *Oligaphorura groenlandica* described by Tullberg from Greenland and Svalbard probably belongs to the main parthenogenetic form with circumpolar distributional range lacking cuticular fold on the sternum of *Abd*.4.

#### Oligaphorura ursi

Taxon classificationAnimaliaCollembolaOnychiuridae

(Fjellberg, 1984)

Onychiurus ursi Fjellberg, 1984: 71.
Oligaphorura ursi (Fjellberg): www.collembola.org

##### Remarks.

Contrary to *Oligaphorura groenlandica*, *Oligaphorura ursi*, another northern circumpolar species of the genus, is common in the Magadan region inhabiting different wet sites above tree-line. Recently the species was redescribed on the basis of specimens from northern China (Sun and Wu 2012a). We have some doubts about the identity of the Chinese and northern populations. Northern specimens usually have 6 long and four spiniform guard setae on the labial palp [versus 11 in Chinese specimens], ventral *psx* 10/000/212201+1^m^ with frequent variations [versus 0/000/122200 in the Chinese ones] and at least one *psx* on each subcoxae, most usually 1-2-2 [versus completely absent]. Apart from this, it was said that the Chinese specimens had an identical number of setae on all tibiotarsi [versus 20-20-19 setae in northern populations].

#### Oligaphorura aborigensis

Taxon classificationAnimaliaCollembolaOnychiuridae

(Fjellberg, 1987)

Onychiurus (Archaphorura) aborigensis Fjellberg, 1987: 285.
Oligaphorura aborigensis (Fjellberg): www.collembola.org

##### Material.

holotype, ♀, “USSR, Magadan Reg., Aborigen [67°57'N, 149°34'E], alpine snow fields, under stones, 27 vii 1979“ (CNC 165043, type No 20102); paratypes, ♀ and juv., same sample (CNC 165127, type No 20101), all A. Fjellberg leg.

Unfortunately the types of the species were partly damaged and no additional specimens were found in the available material from the vicinity of Aborigen field station. So, only few additional details can be added to the original description.

Labium with thick terminal seta only on papilla *A*, 7 long guard setae and 6 proximal setae, basal fields with 4+6 setae. Tibiotarsi with complete set of setae (20-20-19): each distal whorl (*A+T*) with 11 setae, whorl *B* with 7-7-6 setae, setae *M* and *Y* present on all tibiotarsi. Furcal fold straight and comparatively small, situated in mid-section of *Abd*.4, furcal field in the only seen juvenile with 4+4 setae between proximal *q*-setae and the cuticular fold (as on Fig. 24), adults with some additional setae in intermediate position forming 4 more or less regular rows as typical for other *Oligaphorura*.

**Figures 21–24.**
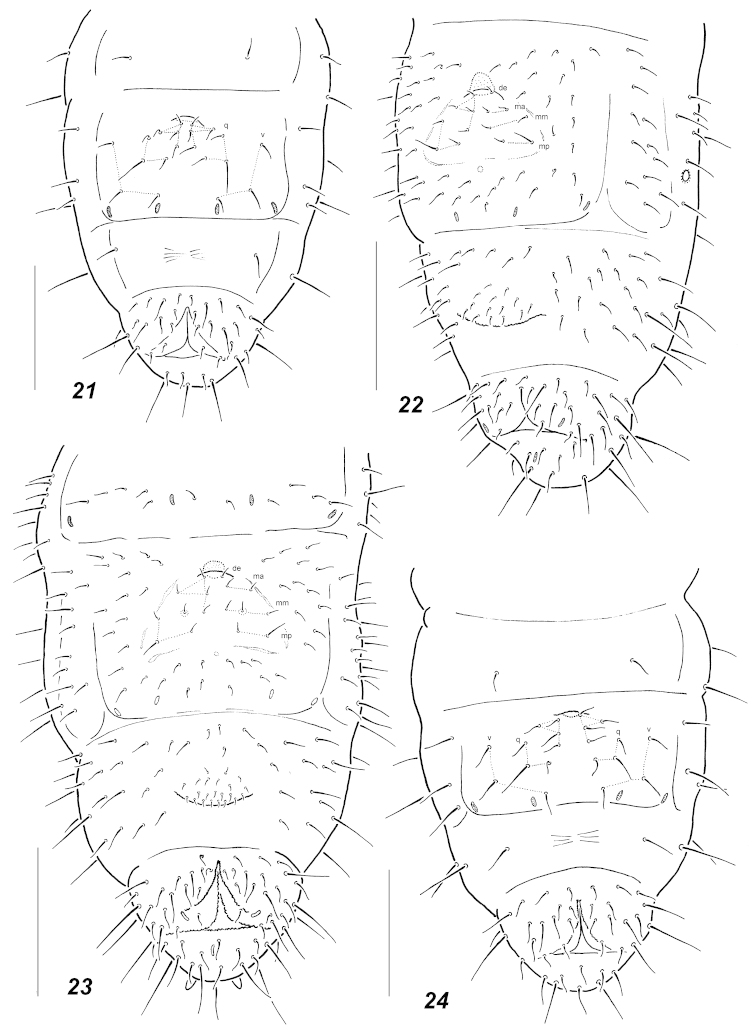
Chaetotaxy of abdominal sterna. **21** *Oligaphorura ursi* (I instar) **22** *Oligaphorura ursi* (adult) **23** *Oligaphorura pingicola* (adult) **24** *Oligaphorura pingicola* (I instar). Secondary setae in adults circled. Scale bars: **21, 24** = 0.05 mm, **22–23** = 0.1 mm.

The species is well defined due to the absence of sublobal setae on the maxillary outer lobe (a unique character for the tribe or even for Onychiurinae), strong differentiation of dorsal setae and the pseudocellar formula (32/133/33353) which is not especially common for the tribe being shared with only *Dimorphaphorura pseudoraxensis* (Nosek & Christian, 1983), *Oligaphorura sabulosa* Babenko, 2008, and *Dimorphaphorura jingyueensis* Sun & Wu, 2012. All of them have the usual two sublobals on the maxillary palp and the macrosetae being much shorter and finer than in *Oligaphorura aborigensis*. Apart from this the two former species are characterized by the absence of *ms* on *Th*.3.

#### Oligaphorura pingicola

Taxon classificationAnimaliaCollembolaOnychiuridae

(Fjellberg, 1987)

Onychiurus (Archaphorura) pingicolus Fjellberg, 1987: 285.
Oligaphorura pingicola (Fjellberg): www.collembola.org

##### Material.

holotype, ♂, “Alaska, Prudhoe Bay, *Dryas*-turf on pingo, 16 viii 1976” (CNC 165048, type No 20099); paratypes, 5♀ and ♂, same sample (CNC 165139, type No 200100), all A. Fjellberg leg.

##### Additional material.

50 specimens, Russia, Yakutia (Sakha Republic), Suntar-Khayata Mt. Range, upper reaches of Kyubyume River [63°13'N, 139°32'E], various sites, viii 2002, O. Makarova leg.; 25 specimens, Magadan District, upper reaches of Ola River [60°39'N, 151°16'E], snow fields, 1100–1200 m alt., A. Babenko leg.; 4 specimens, Magadan District, Bolshoi Annachag Mts. Range, upper reaches of Kolyma River, field station “Aborigen” [61°56'N, 149°40'E], thick moss among rocks near snow field, 26 vii 1979, A. Fjellberg leg.; 4 specimens, same region, lichen/*Ledum* in northern slope, 28 vii 1979, A. Fjellberg leg.; 1 specimens, same region, *Pinus pumila* and lichen cover on hillside, 20 vii 1979, V. Behan leg.; 2 specimens, same region, alpine study area (lichen, moss, *Dryas*, *Empetrum*), 26 vii 1979, V. Behan leg.

##### Redescription.

Colour white. Granulation distinctly enlarged on *Abd*.6 and on head. Size 1.0–1.1 mm. Body shape cylindrical. Antennae about as long as head, *Ant*.3-4 broad, club-like. *Ant*.4 with a subapical spherical organite and a microsensillum located in proximal row of setae. *AO* consists of 5 long and thin papillae, two sensory rods, two granulated sensory clubs (internal almost straight, external much larger and bent), 5 guard setae and a lateral microsensillum which set below the organ. *Ant*.1 and 2 with 8-9 and 15-16 setae respectively. *PAO* with 2-3 elongated lobes, much longer than nearest pseudocellus. Labrum with 4/5-2-2 setae. Apical part of labium with thick terminal setae on papillae *A* and *C*, 6 long guard setae and 6 proximal setae present, basal fields with 4+(5)6 setae. Maxillary palp simple with two sublobal hairs.

Pseudocellar formula (*pso*) as follows, dorsal: 32/033/33343, ventral: 2/000/0000, parapseudocelli (*psx*) 10/000/222201+1^m^. Each subcoxa with one *pso* and one *psx*, *psx* present also on femora and on border between *Ant*.3-4. Granulation rather fine but clearly coarser around pseudocelli and on *Abd*.6. Dorsal chaetotaxy almost symmetrical, setae smooth and clearly differentiated, sensory setae more or less distinct, usually 2/011/222211. *Th*.1 with 7-8 setae on each side, both *Th*.2 and 3 with lateral *ms*, *p*_1_ on head and *Th*.2-3 usually moved forward in relation to other medial *p*-setae. *Abd*.5 with microsetae *m*_1_ thin and curved, clearly shorter than mesosetae *a*_1_ and *p*_1_. Unpaired seta *d*_0_ on head absent, *Abd*.5 frequently with one unpaired axial seta in *p*-row, two axial setae present on *Abd*.6, *a*_0_ shorter than *a*_2_. Thoracic sterna of both *Th*.2 and 3 with 1+1 setae along ventral line. Furca shaped like a small fold in some distance from anterior border of *Abd*.4, in juveniles furcal field with 4+4 setae between proximal *q*-setae and the cuticular fold (Fig. 24), adults with some additional setae in intermediate position forming 4 more or less regular rows as typical for *Oligaphorura* (Fig. 23). Ventral tube with 8-9 distal and (1)2 proximal setae at base. Subcoxae with 4(5-6)-5(6)-5(6) setae, tibiotarsi with 20-20-19 setae: each distal whorl (*A+T*) with 11 setae, whorl *B* with 7-7-6 setae, setae *M* and *Y* present on all tibiotarsi. Unguis without inner tooth but usually with small and hardly visible lateral teeth present, unguiculus with small basal lamella about 3/4 as long as unguis. Anal spines bent, rather thick, set on low papillae. Males present.

##### Remarks.

The above redescription is in full accordance with the original one, adding a few details. While originally described from Alaska, Fjellberg (1987) also remarked that two specimens of the main form are also seen from alpine meadows at Aborigen, USSR (*Magadan Reg*.). In fact the species seems to be widespread and common not only in the Magadan Region but also in inner parts of the eastern Palaearctic (Suntar-Khayata Mts. Range, Yakutia). Fjellberg (1987) mentioned two distinct forms for Alaska differing in mutual position of setae on *Abd*.5 and in level of granulation. Only the main form seems to be present in the eastern Palaearctic.

*Oligaphorura pingicola* shares the number of dorsal and ventral pseudocelli with at least five known species of the tribe, namely *Oligaphorura koreana*, *Oligaphorura nataliae*, *Dimorphaphorura raxensis*, *Dimorphaphorura chatyrdagi*, and *Dimorphaphorura sanjiangensis*. *Oligaphorura koreana* is very similar to *Oligaphorura pingicola*, differing by fewer tibiotarsal setae (19-19-18 versus 20-20-19) and by absence of *psx* (“indistinct”). The absence of *psx* is also characteristic for *Oligaphorura nataliae* which differs from *Oligaphorura pingicola* in having 7 long guard setae on labial palp and absence of *ms* on *Th*.3, as well as 2 setae of the proximal row on labrum (4/7 as a whole). *Dimorphaphorura raxensis* has 9 distal setae on tibiotarsi, full number of labral setae and *ABC* type of labium (Weiner and Kaprus’ 2014). *Dimorphaphorura chatyrdagi* can easily be distinguished due to reduced tibiotarsal chaetotaxy with only 5 distal setae, whereas *Dimorphaphorura sanjiangensis* apart from the chaetotaxy of the sternum of *Abd*.4 differs in labium type (*A* versus *AC*) and identical number of tibiotarsal setae on all legs (20-20-20 versus 20-20-19).

*Oligaphorura tottabetsuensis* (Yosii, 1972), a species known from northern Japan, probably also belongs to the same group although the reported number of dorsal pseudocelli is slightly different (32/033/33333). The species is in need of redescription.

## Discussion

The morphological characters being widely accepted as separating genera of Onychiuridae involve the shape of the postantennal organ (*PAO*), structures of the antennal organ (*AO*), tibiotarsal chaetotaxy, arrangement of the pseudocelli (*pso*), presence/absence of anal spines, distribution and shape of sensory setae on the body, and the gradual reduction of the furca. In our view a genus diagnosis based exclusively on reductional stages of the furca is dubious for at least two reasons: (1) similarity in reductional stage may represent a convergence achieved independently from distant phyletic lines, resulting in a polyphyletic or paraphyletic assemblage of species; (2) many collembolan genera (*Xenylla*, *Folsomia*, *Folsomides*, *Scutisotoma*, etc.) cover species with a wide range of furcal reduction, but are still accepted as natural genera which no one would split. In Collembola at least the initial stages of furcal reduction are clearly of adaptive nature, reflecting a shift from surface activity to life in deeper strata where jumping ability is restricted. Although the species under discussion have a furca which is no longer functional, the adaptive character of the reduction probably masks the underlying genetic relationships. Moreover, the practically identical furcal remnant of the Onychiuridae genera *Supraphorura* Stach, 1954 and *Psyllaphorura* Bagnall, 1948 is obviously not a good proof for any close relationship.

Bagnall’s (1949) original diagnoses of four genera of Oligaphorurini were more species than genus diagnoses. Re-establishment of these genera by Weiner (1996) and Pomorski (1996) was based on other diagnostic characters and involved more species but created some taxonomic problems which are not yet solved. According to these authors the four principal genera may be recognized as follows: *Archaphorura* and *Oligaphorura* differ by absence of anal spines in the former, presence in the latter. Both have identical furcal fields, differing from the two other genera by an additional row of setae, even in the first instar juvenile. *Dimorphaphorura* has the same chaetotaxy of the furcal field as a juvenile *Micraphorura*, whereas adults of the latter have a few (1-4) additional intermediate setae. In practice juveniles of *Archaphorura* and *Oligaphorura* are easily separated, also from juveniles of *Dimorphaphorura*/*Micraphorura*, while juveniles of the two latter are inseparable by the furcal field. Sorting out the generic affiliation of adults is much more difficult.

On the www.collembola.org there is an interactive key which proposes the following characters for identification of Oligaphorurini genera.

*Chribellphorura*: antennal tip with a retractive papilla, tibiotarsi with clavate setae in distal whorl; *Archaphorura*: *Abd*.5-6 fused dorsally, *Ant*.3-4 fused, anal spines absent; *Dimorphaphorura*: furcal rudiment in a form of finely granulated area; *Oligaphorura*: furcal rudiment in a form of cuticular furrow or small fold; chaetotaxy: 2 dental setae on the fold or posteriorly and three manubrial rows of setae behind them; *Micraphorura*: similar to *Oligaphorura* but without 2 dental setae, so only three rows of setae can be distinguished, *mm*-row with 4-6 setae.

In summary, *Archaphorura* has a unique character combination, *Dimorphaphorura* has no furcal fold or furrow, and *Oligaphorura* has an additional row of setae on the manubrial field compared with *Micraphorura*. The monotypic genus *Chribellphorura* is unique and needs no further discussion to be distinguished.

This adequate but probably too simplified scheme was neglected by Shvejonkova and Potapov (2011) who included three new species without anal spines and furcal fold not in *Archaphorura* but in *Micraphorura* (*Micraphorura stojkoae*) and *Oligaphorura* (*Oligaphorura kremenitsai* and *Oligaphorura humicola*). As a result *Archaphorura* lost its main diagnostic feature (absence of anal spines), as *Dimorphaphorura* (furcal rudiment in the form of a finely granulated area). The authors considered *Archaphorura* to be a good genus not due to the absence of *AS*, but because of the peculiar antennae (fused *Ant*.3 and 4, subapical position of *AO* and *ms* of *Ant*.4 hidden under long papillae) and the fused *Abd*.5-6. There is also one neglected diagnostic character state of *Archaphorura serratotuberculata*, the type species of the genus, namely the absence of *M*-setae on tibiotarsi. This very character was registered in two rather remote European regions, Fennoscandia (Fjellberg 1998) and Moscow vicinity (new data). In all other Oligaphorurini with known tibiotarsal chaetotaxy this seta is present. Unfortunately there is still a number of species in which tibiotarsal chaetotaxy is not completely known and the character may end up as non-diagnostic for *Archaphorura*. The exact definition of *Archaphorura serratotuberculata* is obscure (Shvejonkova and Potapov 2011) and several species may be involved.

The recent revision of the Palaearctic species of *Dimorphaphorura* by Weiner and Kaprus’ (2014) defined the genus more legibly and added a further criterion distinguishing *Dimorphaphorura* from other genera of the tribe – the absence of so called “dental” setae. In our view this character is rather subjective as setae set just below cuticular trace of furca differ (in size or sockets) from other sternal setae on *Abd*.4 only occasionally even in *Micraphorura absoloni*, can hardly represent a reliable criterion. The two new species described in the present paper further complicate the situation as one of them lacks *AS* but has long, not club-like, antenna and both have “furca” in the form of a “finely granulated area” (typical of *Dimorphaphorura*) but with 2+2 small setae in two rows below it as in *Oligaphorura* (Figs 15–16, 29–30).

In fact the diversity of manubrial chaetotaxy patterns in Oligaphorurini seems to be much higher than postulated so far, which obscures the current generic subdivisions. Thus four different patterns were found in juveniles of the northern species of the tribe: apart from 3+3 proximal *q*-setae the furcal field may have 2+2 setae (*alnus*, *absoloni*, Fig. 27), 3+3 (*groenlandica*, *nataliae*, *interrupta*, Figs 18–19, 32) or 4+4 setae (most studied *Oligaphorura*, Figs 21, 24). In the latter case there are at least two patterns with a different mutual position of setae (*cf.* Fig. 21 and Fig. 26). The fifth variant with only 1+1 setae is known for the first instar of *Dimorphaphorura daii* (Pomorski et al., 1998). The number of secondary setae appearing during ontogenesis on the furcal field is surprisingly low, usually 1-3, rarely more. Nevertheless, the position and the number of these secondary setae are not stable within a species. We have probably never seen any adult specimens with completely symmetrical chaetotaxy of furcal field when there are any secondary setae present.

**Figures 25–28.**
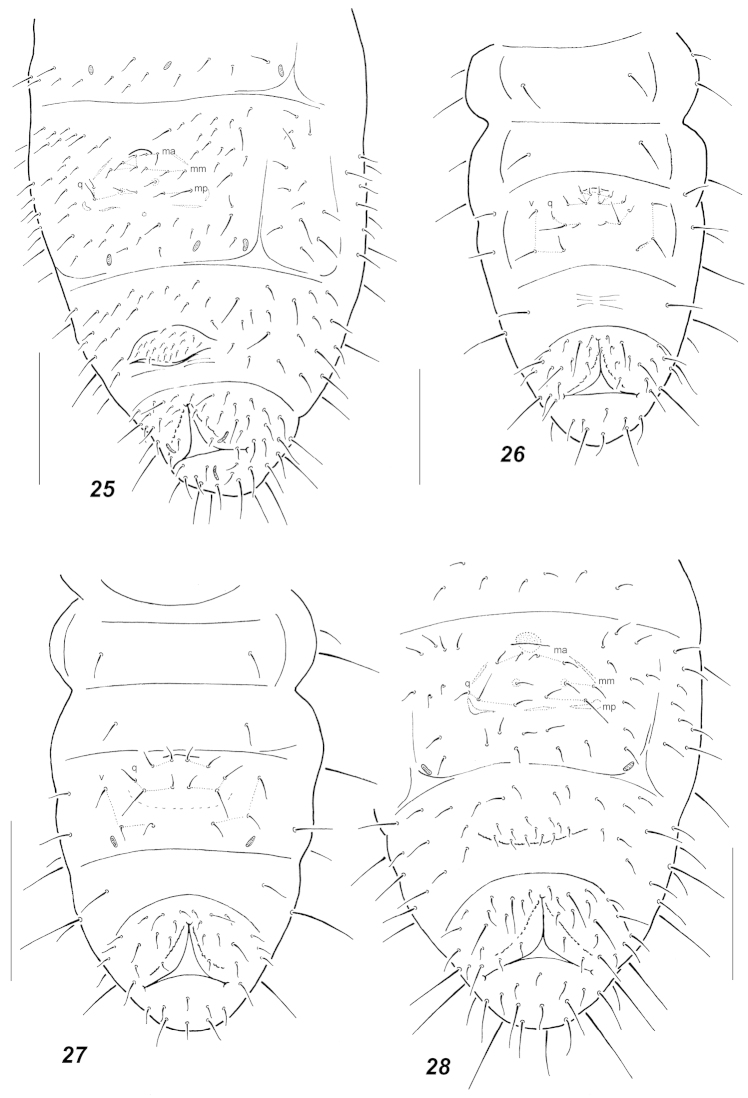
Chaetotaxy of abdominal sterna. **25** Oligaphorura sp. aff. groenlandica (adult, Taimyr) **26** Oligaphorura sp. aff. groenlandica (I instar, Taimyr) **27** *Micraphorura absoloni* (I instar) **28** *Micraphorura absoloni* (adult). Secondary setae in adults circled. Scale bars: **9** = 0.1 mm, **10–12** = 0.05 mm.

**Figures 29–32.**
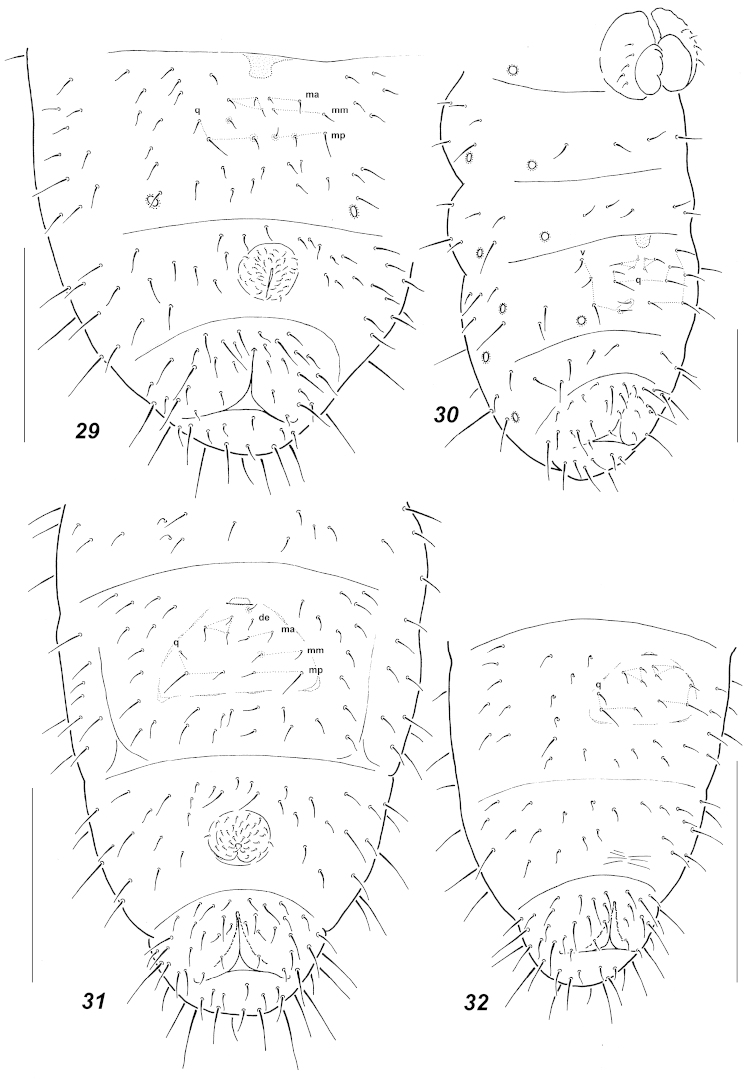
Chaetotaxy of abdominal sterna. **29** *Oligaphorura ambigua* sp. n. (adult) **30** *Oligaphorura ambigua* sp. n. (juvenile) **31** *Oligaphorura nataliae* (adult) **32** *Oligaphorura nataliae* (juvenile). Secondary setae in adults circled. Scale bars: **29, 31–32** = 0.1 mm, **30** = 0.05 mm.

**Figures 33–34.**
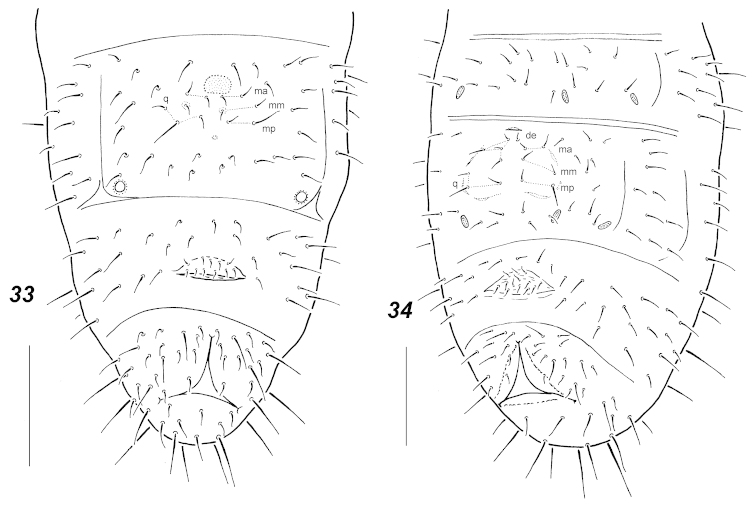
Chaetotaxy of abdominal sterna. **33** *Micraphorura alnus* (adult) **34** *Archaphorura serratotuberculata* (adult). Secondary setae circled. Scale bars: 0.05 mm.

The present generic framework for Oligaphorurini is probably unique – difficult to use and hardly reflecting real relationships. There is great temptation to return to a single genus *Archaphorura* as Christiansen and Bellinger (1980, 1998) and Fjellberg (1987) had done. Nevertheless we realize that such a pooling of all species of the tribe within a single unit obviously contradicts recent taxonomic traditions. Despite being unsatisfactory the most realistic alternative is to keep the current “five genera” system, admitting that this solution is clearly provisional and does not fully reflect a natural generic affiliations of the species pool. Hopefully future genetic studies (barcoding) may contribute to solve these problems.

### Key to the northern species of the Oligaphorurini tribe

**Table:** 

1	*Th*.1 without *pso*	**2**
–	*Th*.1 with *pso*	**6**
2	Only *Th*.2 with lateral *ms*	***Oligaphorura nataliae* (Fjellberg)**
–	Both *Th*.2 and *Th*.3 with lateral *ms*	**3**
3	*Abd*.4 with 4 dorsal *pso* [totally 32/033/33343]	***Oligaphorura pingicola* (Fjellberg)**
–	*Abd*.4 with 5 dorsal *pso* including 3 in submedial group	**4**
4	*Th*.2 with at least 1+1 ventral setae. *Abd*.1-3 with *pso b* moved forward above medial *pso a*. Dorsal *pso* formula as 32/033/44454	***Oligaphorura reversa* (Fjellberg)**
–	*Th*.2 without ventral setae. *Abd*.1-3 with *pso b* set behind medial *pso a*	**5**
5	*Th*.3 at least sometimes with 1+1 ventral setae	***Oligaphorura interrupta* (Fjellberg)**
–	*Th*.3 without ventral setae	***Oligaphorura nuda* (Fjellberg)**
6	*Th*.1 with 2+2 *pso* and with only 4+4 setae present in adults. Thoracic sterna without setae	***Oligaphorura duocellata* sp. n.**
–	*Th*.1 with only 1+1 *pso* and more than 4+4 setae in adults. *Th*.2-3 as a rule with ventral setae	**7**
7	Only *Th*.2 with lateral *ms*	8
–	Both *Th*.2 and *Th*.3 with lateral *ms*	**11**
8	*AO* with 4 papillae	**9**
–	*AO* with 5 papillae	**10**
9	Anal spines present. Upper subcoxae with 1-1-1 *pso*. Labrum with 9 setae. Labium of *AC* type	***Micraphorura absoloni* (Börner)**
–	Anal spines absent. Upper subcoxae with 2-(2)3-3 *pso*. Labrum with 7 setae. Labium of *ABC* type	***Oligaphorura ambigua* sp. n.**
10	*Abd*.4 with 4 dorsal *pso* [totally 32/133/33343]. Labium *A*-type	***Oligaphorura ursi* (Fjellberg, 1984)**
–	*Abd*.4 with 5 dorsal *pso* [totally 32/133/33353]. Labium *AC*-type	***Oligaphorura sabulosa* Babenko, 2008**
11	Maxillary outer lobe without sublobals. Formula of dorsal *pso* as follows 32/133/33353	***Oligaphorura aborigensis* (Fjellberg)**
–	Maxillary outer lobe with 2 sublobals. Formula of dorsal *pso* as follows 32/133/33343	**12**
12	Labium of *A*- type	***Oligaphorura schoetti* (Lie-Pettersen)**
–	Labium of *AC* type	**13**
13	*Abd*.4 with ventral *pso*. Labrum with 7 setae	***Micraphorura alnus* (Fjellberg)**
–	*Abd*.4 without ventral *pso*. Labrum with 9 setae	***Oligaphorura groenlandica* (Tullberg)**

## Supplementary Material

XML Treatment for Oligaphorura ambigua

XML Treatment for Oligaphorura duocellata

XML Treatment for Micraphorura absoloni

XML Treatment for Oligaphorura nataliae

XML Treatment for Micraphorura alnus

XML Treatment for Oligaphorura interrupta

XML Treatment for Oligaphorura groenlandica

XML Treatment for Oligaphorura ursi

XML Treatment for Oligaphorura aborigensis

XML Treatment for Oligaphorura pingicola
